# Mesenchymal Stem Cell Conditioned Medium Modulates Inflammation in Tenocytes: Complete Conditioned Medium Has Superior Therapeutic Efficacy than Its Extracellular Vesicle Fraction

**DOI:** 10.3390/ijms241310857

**Published:** 2023-06-29

**Authors:** Robert Soukup, Iris Gerner, Thomas Mohr, Sinan Gueltekin, Johannes Grillari, Florien Jenner

**Affiliations:** 1 VETERM, Equine Surgery Unit, Department for Companion Animals and Horses, Vetmeduni, 1210 Vienna, Austriairis.gerner@vetmeduni.ac.at (I.G.);; 2 Austrian Cluster for Tissue Regeneration, 1200 Vienna, Austria; 3 Science Consult DI Thomas Mohr KG, 2353 Guntramsdorf, Austria; 4 Center for Cancer Research, Comprehensive Cancer Center, Medical University of Vienna, 1090 Vienna, Austria; 5 Department of Analytical Chemistry, University of Vienna, 1090 Vienna, Austria; 6 Ludwig Boltzmann Institute for Traumatology, The Research Center in Cooperation with AUVA, 1200 Vienna, Austria; 7 Institute of Molecular Biotechnology, University of Natural Resources and Life Sciences, 1090 Vienna, Austria

**Keywords:** equine mesenchymal stem cells (MSC), MSC secretome, MSC extracellular vesicles (EVs), MSC protein fraction, cell-free therapy, tenocytes regeneration

## Abstract

Tendinopathy, a prevalent overuse injury, lacks effective treatment options, leading to a significant impact on quality of life and socioeconomic burden. Mesenchymal stem/stromal cells (MSCs) and their secretome, including conditioned medium (CM) and extracellular vesicles (EVs), have shown promise in tissue regeneration and immunomodulation. However, it remains unclear which components of the secretome contribute to their therapeutic effects. This study aimed to compare the efficacy of CM, EVs, and the soluble protein fraction (PF) in treating inflamed tenocytes. CM exhibited the highest protein and particle concentrations, followed by PF and EVs. Inflammation significantly altered gene expression in tenocytes, with CM showing the most distinct separation from the inflamed control group. Treatment with CM resulted in the most significant differential gene expression, with both upregulated and downregulated genes related to inflammation and tissue regeneration. EV treatment also demonstrated a therapeutic effect, albeit to a lesser extent. These findings suggest that CM holds superior therapeutic efficacy compared with its EV fraction alone, emphasizing the importance of the complete secretome in tendon injury treatment.

## 1. Introduction

Tendinopathy, a disabling overuse injury, is prevalent among athletes and sedentary subjects, afflicting 25% of the adult population and accounting for 30–50% of all sports injuries [1,2]. No current treatment restores the functional properties of injured tendons, resulting in a significant impact on quality of life and high socioeconomic pressure, with the annual health expenditure on human tendon injuries exceeding €145 billion.

The repair response of tendons following injury is inefficient, resulting in a fibrovascular scar that never attains the gross, histological, or mechanical characteristics of normal tendon and thus predisposes to recurring injury and tendinopathy [1,2,3,4,5,6,7,8]. Tendon injury induces a local inflammatory response, characterized by an influx of inflammatory cells that release chemotactic and proinflammatory cytokines and growth factors, promote the recruitment and proliferation of macrophages and resident tendon fibroblasts, and initiate the healing process [3,4,5,6,7,8,9,10,11,12]. The inflammatory milieu can modify tenocyte physiology by increasing metabolic activity and inducing an activated, proinflammatory phenotype with inflammation memory and the capacity for endogenous production of inflammatory cytokines such as TNF-α and IL-1β [12]. There is growing evidence that inflammation (the first phase of the injury response), specifically its lack of resolution, has a crucial role in disease progression, especially when shifting to a chronic state [3,4,5,6,7,8,9,10,11,12].

Tendon treatment with mesenchymal stem/stromal cells (MSCs) has been employed in equine orthopaedics since 2003 and has yielded promising results, reducing reinjury rates in the equine superficial digital flexor tendon, the functional equivalent to the human Achilles tendon, from 56% to 18% [13,14,15,16,17,18,19,20]. Yet, translational progress into human clinical practice so far has been disappointing, partially due to the regulatory and manufacturing challenges inherent to all cell therapies and safety concerns such as potential tumorigenicity [21]. In addition, inflammatory conditions can compromise MSC’s differentiation capacity and therapeutic potential, as MSCs react context-sensitively to their respective pathophysiological microenvironments [22]. However, MSCs show poor engraftment and cell survival following transplantation and exert their therapeutic effect predominantly by secreting bioactive factors (collectively termed “secretome”), including soluble factors and extracellular vesicles (EVs) with their cargo of proteins, lipids, metabolites, and nucleic acids [23,24,25]. The secretome mirrors the ability of the parental cells to condition and program the surrounding microenvironment, influencing a variety of endogenous responses, in particular in injured tissues. Indeed, both the whole secretome (or conditioned medium, CM) and its EV fraction have shown equivalent therapeutic effects on their producer cells in a wide variety of diseases, including spinal cord injury, cardiomyopathy, osteoarthritis, and tendinitis, thus paving the way for the development of cell-free therapies [23,24,26,27,28,29,30,31,32,33].

MSC-CM and EVs have shown immunomodulatory effects by modulating macrophage polarization toward a pro-resolving M2 phenotype, reducing the expression of pro-inflammatory cytokines such as TNFα, interleukin (IL)-1β and IL-6 and increasing the secretion of anti-inflammatory factors including IL-10 and IL-4 [34,35,36]. In addition, MSC-CM and EVs contribute directly to tissue regeneration by enhancing tissue-specific extracellular matrix (ECM) component production and downregulating catabolic matrix metalloproteinases (MMPs) [35,36].

The regenerative potential of MSC-CM and EVs in tendon and ligament repair was shown in vitro and in vivo in various species. including mice, rats, rabbits, sheep, horses, and humans [34,37,38,39,40,41,42,43,44,45,46,47,48]. MSC-derived CM (MSC-CM) promoted rat tenocyte proliferation via activation of extracellular signal-regulated kinase1/2 (ERK1/2) signal molecules compared with the untreated control group [49]. Similarly, MSC-CM promoted tendon-bone healing of the rat rotator cuff by inhibiting M1 and supporting M2 macrophage polarization [34]. Furthermore, in horses, MSC-CM demonstrated an immunomodulatory effect by inhibiting the proliferation of peripheral blood mononuclear cells (PBMCs) in vivo and induced neovascularization, which was not observed before treatment and declined during the progression of the healing process, characterized by a decrease in vessel size and quantity [50]. MSC-EVs demonstrated their immunomodulatory capacity in a variety of tendon injury models in vivo by reducing macrophage NF-κB activity and the IL-1β and IFN-γ response, decreasing M1 and supporting M2 macrophage polarization, and increasing the production of anti-inflammatory cytokines such as IL-4 and IL-10 [37,43,45,47]. In addition, MSC-EVs administered into tendon and enthesis defects resulted in enhanced proliferation of tendon stem/progenitor cells (TSPCs), better restoration of the tendon and enthesis architecture, an improved histological score, greater expression of genes related to collagen and tendon matrix formation, including collagen (Col) type I, mohawk (MKX), scleraxis (SCX)tenomodulin (TNMD) and tissue inhibitor of metalloproteinase-3 (TIMP-3), and decreased matrix metalloproteinases (MMP)-3 expression [37,38,40,44,46].

While the therapeutic potential of MSC-CM and its EV fraction for regenerative medicine in general and the treatment of tendon injuries in particular is well established, it is still unclear whether components of the CM act synergistically or redundantly and whether the entire CM, isolated EVs, or only selected soluble factors are required to achieve a therapeutic effect [51,52]. Attempts to use single paracrine factors, such as beta fibroblast growth factor (bFGF), hepatocyte growth factor (HGF), and vascular endothelial growth factor (VEGF), to treat cardiovascular diseases so far have shown unsatisfactory outcomes, demonstrating that these factors alone are not sufficient to induce regeneration [52]. A study comparing the therapeutic efficacy of complete MSC-CM and MSC-EVs alone on skeletal muscle regeneration in vitro and in vivo demonstrated that the two fractions promote different aspects of regeneration after muscle injury and act synergistically to promote muscle regeneration [53]. In detail, CM but not the EV fraction inhibited cellular senescence, but only EVs impacted nuclear translocation of NF-κB and decreased lysosomal activity in glioma cells. Similar results were obtained in a wound closure model, suggesting that the complete CM has superior regenerative capacity than its purified subfractions [54].

Therefore, this study compares the therapeutic effect of CM, its EV fraction, and its soluble protein fraction (PF, CM without EVs) on inflamed tenocytes to test the hypothesis that the conditioned medium has greater therapeutic efficacy than its EV fraction alone. The study is carried out in equine cells, as horses suffer from naturally occurring tendinopathy analogous to humans and are well-established animal models, also recommended by the U.S. Food and Drug Administration (FDA) and the European Medicines Agency (EMA) [55,56,57].

## 2. Results

### 2.1. Equine Bone Marrow-Derived MSCs and EVs Show Characteristic Markers

**Figure 1:**
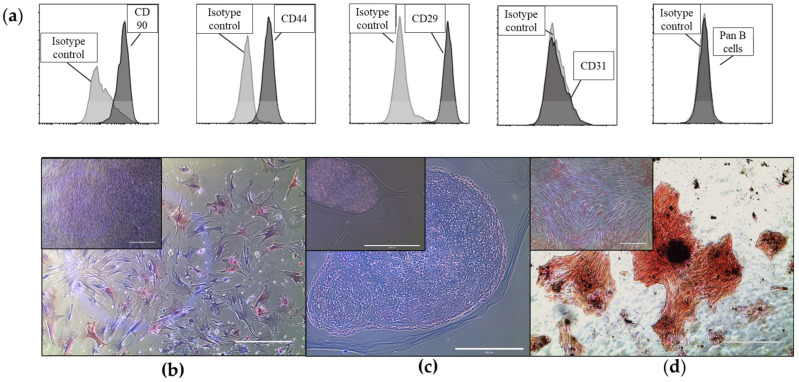
Shows representative images of the characterization of equine bone-marrow-derived cells with trilineage differentiation capacity and canonical surface marker expression of MSCs. (**a**) Cells were stained with specific cell surface antigens (dark grey plots) or Ig isotype controls (light grey plots) and analyzed by flow cytometry. Cells stained positive for CD90, CD44, and CD29 and negative for CD31 and Pan B, as well as IgG isotype controls. Displayed on the *x*-axis is either PE or FITC conjugated to one of the previously mentioned antibodies. (**b**–**d**) Images show bone-marrow-derived cells differentiating into the adipogenic (**b**), chondrogenic (**c**), and osteogenic (**d**) lineages, stained with Oil red O, Alcian blue, and von Kossa stain, respectively. The corresponding controls (cells grown in an expansion medium) are shown in the insert in the top left corner of each micrograph. Scale bars: 400 µm.

The cells isolated from the bone marrow of the three donor horses were plastic adherent and expressed typical MSC markers such as CD90 (73.1% ± 5.3 positive cells), CD44 (77.2% ± 3.1 positive cells), and CD29 (72% ± 3.7 positive cells) (Figure 1a) [58]. They exhibited the ability to differentiate into adipocytes (Figure 1b), chondrocytes (Figure 1c), and osteoblasts (Figure 1d), demonstrating their trilineage differentiation potential [58]. We characterized the EVs according to the MISEV2018 guidelines using nanotracking analysis, fluorescence-triggered flow cytometry (lipid membrane dye cell mask green (CMG) and CD81), Western Blot (positive for the two tetraspanins, CD9 and CD63), and transelectron microscopy as we previously described [58].

### 2.2. CM Fraction Has the Highest Protein- and Particle Concentration

**Figure 2:**
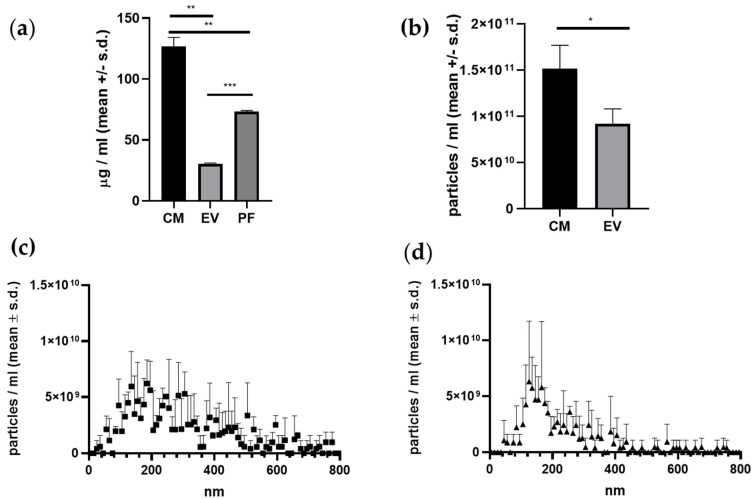
MSCs from 3 equine donors were cultured and the cell-free supernatant was collected and centrifuged to obtain CM, EVs and PF. (**a**) shows that the protein concentration was significantly higher in the CM fraction than in the EV and PF fractions (** = *p* = 0.0019 and *** = *p* = 0.0066, respectively). Moreover, the protein concentration was higher in the PF fraction than in the EV fraction (*** = *p* = 0.0005). (**b**) shows that the particle concentration was significantly higher in the CM fraction than in the EV fraction (* = *p* = 0.0179). (**c**,**d**) illustrate the size distribution of the EVs and CM, respectively. The EV fraction contained more particles ≤195 nm, resulting in a more homogeneous size distribution compared with the CM fraction.

After CM production for 48 h, the measured protein concentration was highest in the CM fraction, with a mean of 130 ± 6.2 µg/mL (Table 1). The protein concentration in the CM fraction was 4.3 times higher than in the EV fraction (30.4 ± 0.5 µg/mL) (*p* = 0.0019) and 1.8 times higher than in the PF fraction (73.2 ± 0.9 µg/mL) (*p* = 0.0066). The PF fraction contained 2.4 times more protein than the EV fraction (*p* = 0.0005) (Figure 2a and Table 1).

Particle concentration was also higher in the CM fraction compared with the EV fraction, with 1.53 × 10^11^ ± 2.01 × 10^10^ particles/mL in the CM fraction and 1.03 × 10^11^ ± 2.04 × 10^10^ particles/mL in the EV fraction (Figure 2b). No particles were detected in the PF fraction. The size distribution of the particles was more homogeneous in the EV fraction, with 5.61 × 10^10^ ± 1.06 × 10^10^ particles/mL particles ≤ 195 nm and 4.67 × 10^10^ ± 9.81 × 10^9^ particles/mL particles > 195 nm. In the CM fraction, 5.36 × 10^10^ ± 7.67 × 10^9^ particles/mL were ≤195 nm while 1.09 × 10^11^ ± 2.51 × 10^10^ particles/mL were >195 nm (Figure 2c,d and Table 1).

There was no significant variation in protein content, particle concentration, or size distribution among the donors.

### 2.3. Inflammation Induces a Significant Change in Tenocytes’ Gene Expression

**Figure 3:**
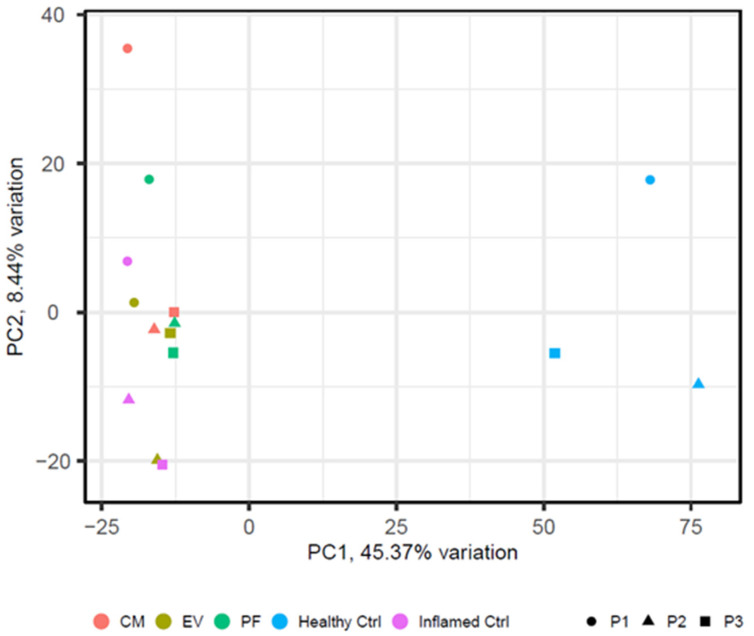
Principal Component Analysis (PCA) plot displaying the differential gene expression data from patient cells. Three patients (P1, P2, and P3) are represented by different symbols, while the treatments and controls are distinguished by different colors. The healthy control (light blue) is separated from the other samples. Cells treated with CM (red dots), inflamed control (purple dots), EV-treated cells (green dots), and PF-treated cells (light green dots) cluster close to each other.

The induction of inflammation was induced by serum starvation plus chemical stimulation with 10 ng/mL TNFα and 10 ng/mL ILb1 for 24 h. This resulted in the differential expression of 1496 genes, representing 12.5% of all identified genes (11,925 genes) as compared with the healthy control (Table 2 and Table 3, Figure 3). 346 (23%) were upregulated in the inflamed control compared with the healthy control, and 1150 (78%) were downregulated. The upregulated genes were found to be strongly associated with inflammation, including Colony Stimulating Factor 3 (CSF3), C-C Motif Chemokine Ligand 11 (CCL11), CCL2, CCL20, CCL5, C-X-C Motif Chemokine Ligand 1 (CXCL1), CXCL6, CXCL8, Interleukin 36 Gamma (IL36G), IL6, NF-kappa-B inhibitor alpha (NFKBIA), and Prostaglandin D2 Synthase (PTGDS). Pathway analysis yielded 112 pathways that were downregulated upon inflammation and 98 that were upregulated (Table 3).

All inflamed groups, treated and untreated alike, clustered distantly from the healthy control group in principal component analysis (Figure 3).

### 2.4. Conditioned Medium Achieves a Higher Effect on Differential Gene Expression of Inflamed Tenocytes Than EVs or PF Alone

**Figure 4:**
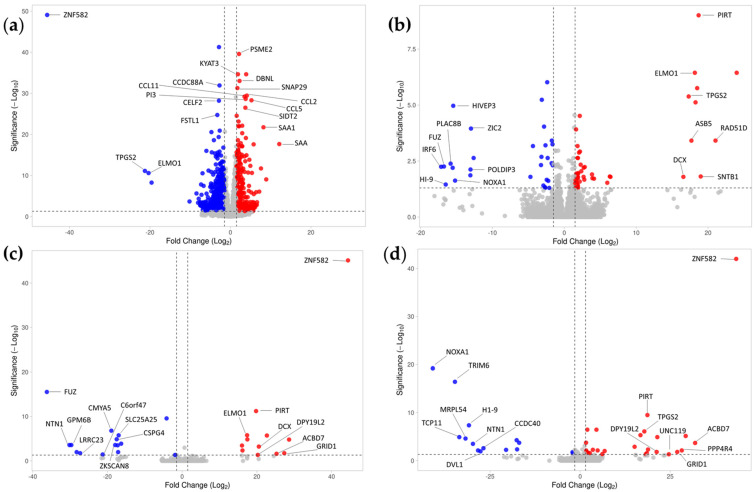
Volcano plot of differentially expressed (DE) genes in the patient cells: The plot highlights the top 20 DE genes, which have been appropriately labeled for identification. (**a**) Control Inflamed vs. Control Healthy (**b**) CM vs. Control Inflamed, (**c**) EV vs. Control Inflamed and (**d**) PF vs. Control Inflamed are displayed in absolute numbers. The volcano plots show the log2 fold change on the *x*-axis and the log10 adjusted *p*-value on the *y*-axis. Significantly upregulated genes are shown in red, significantly downregulated genes in blue, and non-significant genes in grey. Only genes with an adjusted *p*-value of *p* < 0.1 and FC > 1.5 are considered significant.

The tenocytes exhibited a significant differential gene expression as a result of inflammation (Figure 4a). CM had the strongest treatment effect on inflamed tenocytes, resulting in statistically significant differential expression of 120 genes, of which 42 genes (35%) were down-regulated (Table 2) and 78 genes (65%) were up-regulated compared with the inflamed control with no treatment (Figure 4b and Table 3). Treatment with PF had the second strongest effect on gene expression, resulting in the statistically significant differential expression of 57 genes, of which 17 genes (30%) were down-regulated (Table 2) and 40 genes (70%) were up-regulated compared with the inflamed control (Figure 4d and Table 3). Treatment with EV resulted in the statistically significant differential expression of 33 genes, of which 16 genes (48.5%) were down-regulated (Table 2) and 17 genes (51.5%) were up-regulated compared with the inflamed control (Figure 4c and Table 3).

**Figure 5:**
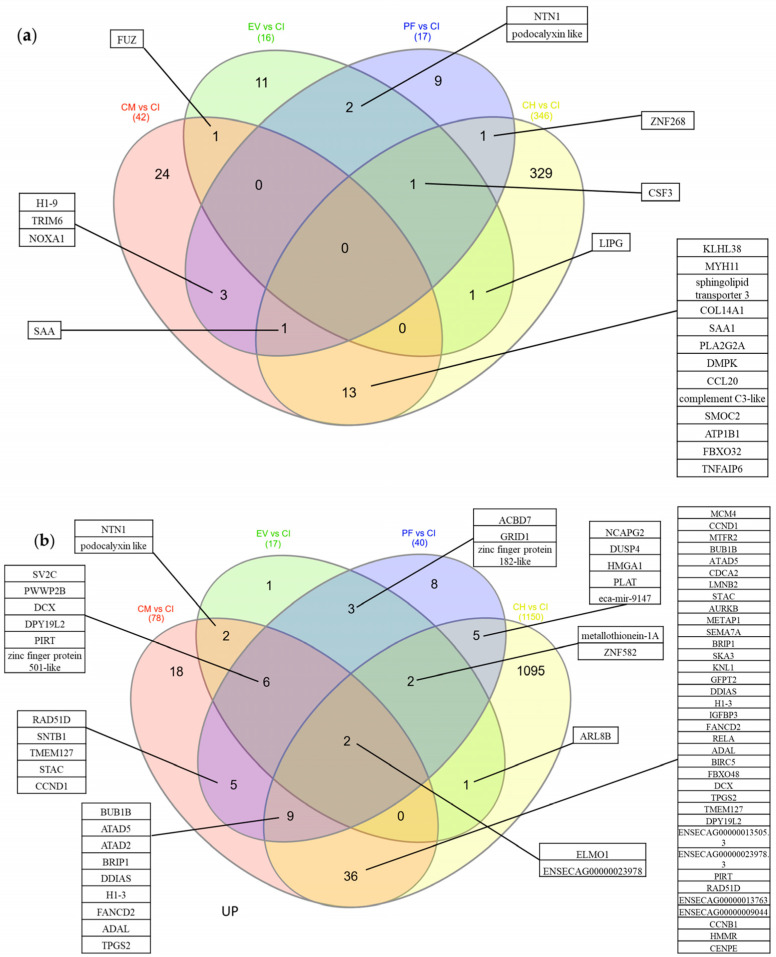
Venn diagram of DE genes in the patient cells: (**a**) Venn diagram of genes which are downregulated upon treatment and in the healthy control compared with inflamed control. (**b**) Venn diagram of genes which are upregulated upon treatment and in the healthy control compared with inflamed control. Only genes with an adjusted *p*-value of *p* < 0.1 and FC > 1.5 are considered significant. In panel (**a**), the Venn diagram shows the number of downregulated genes in the treated cells and healthy control compared with the inflamed control. The number of genes unique to each treatment is displayed in the respective circle, while the overlapping regions represent the number of shared genes between the treatments. Panel (**b**) shows the Venn diagram of upregulated genes in the same comparison. Only genes with an adjusted *p*-value of *p* < 0.1 and FC > 1.5 are considered significant.

CM treatment downregulated 42 genes compared with the untreated inflamed group (Figure 4b and Figure 5a and Table 2). Of these 24 genes (57%) that were unique to CM treatment, 13 genes (31%) were shared with healthy control, three (7%) with PF, one (2%) with EV, and one (2%) with PF and healthy control. The genes that were downregulated following treatment with CM included genes linked to inflammation (CCL20, Serum amyloid A (SAA), SSA1, and Tumor necrosis factor-inducible gene 6 protein (TNFAIP6)) and extracellular matrix organization (Collagen type XIV alpha 1 chain (COL14A1) and Matrix metalloproteinase 13 (MMP13)). In addition, CM treatment upregulated 78 genes compared with the untreated inflamed control, of which 18 (23%) were unique to CM treatment, nine (12%) were shared with PF treatment and healthy control, six (8%) with EV and PF treatment, five (6%) with PF treatment, two (3%) with EV treatment, and two (3%) with EV, PF, and healthy control. The genes that were upregulated upon CM treatment included genes associated with cell cycle and mitosis (various cyclins, kinesin family members, and ATPase family members), DNA damage response and repair (DNA damage-induced apoptosis suppressor (DDIA), Fanconi Anemia Complementation Group D2 (FANCD2), High Mobility Group AT-hook 2 (HMGA2), Platelet-activating factor acetylhydrolase 2 (PAFAH2), V-rel avian reticuloendotheliosis viral oncogene homolog A (RELA)), and metabolism and transport (Glutamine-fructose-6-phosphate transaminase 2 (GFPT2), Methionine Aminopeptidase 1 (METAP1), and Synaptic Vesicle Glycoprotein 2C (SV2C)) (Figure 4b and Table 3).

EV treatment downregulated 16 genes compared with the untreated inflamed group (Figure 4c and Figure 5a and Table 2). Of these 11 genes (69%) that were unique to EV treatment, two (12.5%) genes were shared with PF, one (6.25%) with CM, one (6.25%) with healthy control, and one (6.25%) with PF and healthy control. Among the genes downregulated upon EV treatment were the cytokine CSF3 and glycogen synthase kinase 3 alpha. Furthermore, we observed that EV treatment upregulated 17 genes, of which one (6%) gene was unique to EV treatment, six (35%) were shared with CM and PF, three (18%) genes were shared with PF, two (12%) with CM, two (12%) with PF treatment and healthy control, two (12%) with CM, EV fraction, and healthy control, and one (6%) only with healthy control. The genes that were upregulated upon EV treatment were associated with cytoskeleton organization (ADP Ribosylation Factor Like GTPase 8B (ARL8B) and Doublecortin (DCX)), extracellular matrix organization (Inter-alpha-trypsin inhibitor heavy chain 1 (ITIH1)), and epigenetic modification (Ankyrin Repeat and SOCS Box Containing 5 (ASB5) and Acyl-CoA binding domain containing 7 (ACBD7)) (Figure 4c and Figure 5b and Table 3).

Treatment with PF led to the downregulation of 17 genes compared with the inflamed control (Figure 4d and Figure 5a and Table 2). Of these nine (53%) were unique to PF treatment, three (18%) were shared with CM, two (12%) were shared with EV, one (6%) with EV fraction and healthy control, one (6%) with CM fraction and healthy control, and one (6%) was shared with healthy control. Among the genes downregulated upon PF treatment were genes associated with inflammation (CSF3, IL17RA, and SAA), protein degradation (Tripartite Motif-Containing 6 (TRIM6)), and oxidative stress (NADPH oxidase activator 1 (NOXA1)). Treatment with PF upregulated 40 genes compared with the inflamed control, of which eight (20%) were unique to PF treatment, five (4%) were shared with healthy control, three (7.5%) were shared with EV, two (5%), two (5%) were shared with CM fraction and healthy control, six (15%) were shared with CM, five (12.5%) were shared with CM, and nine (22.5%) were shared with CM fraction and healthy control. The genes that were upregulated upon PF treatment were associated with DNA damage response and repair (BRCA1-Interacting Protein 1 (BRIP1), Fanconi Anemia Complementation Group D2 (FANCD2) and RAD51 Paralog D (RAD51D)), cell cycle regulation (BUB1 Mitotic Checkpoint Serine/Threonine Kinase B (BUB1B), Cyclin D1 (CCND1), Non-SMC Condensin II Complex Subunit G2 (NCAPG2) and Protein Phosphatase 4 Regulatory Subunit 4 (PPP4R4)), cytoskeleton organization (DCX, Dpy-19 Like 2 (DPY19L2), Engulfment and Cell Motility 1 (ELMO1) and Unc-119 Lipid Binding Chaperone (UNC119)), membrane trafficking and fusion (Syntrophin Beta 1 (SNTB1) and Synaptic Vesicle Glycoprotein 2C (SV2C)), and transcriptional regulation (Glutamate Receptor Ionotropic Delta 1 (GRID1), High Mobility Group AT-hook 1 (HMGA1) and Zinc Finger Protein 582 (ZNF582)) compared with the inflamed control (Figure 4d and Figure 5b and Table 3).

### 2.5. Pathways

**Figure 6:**
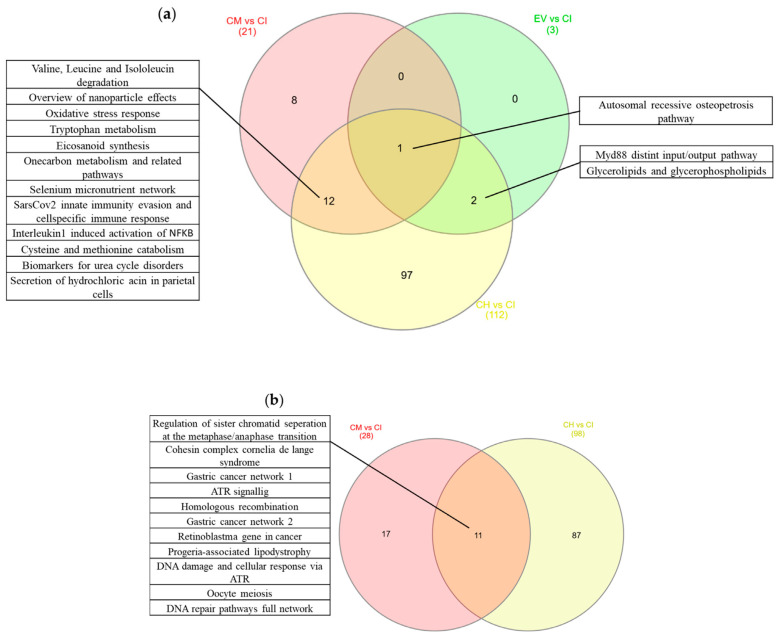
Venn diagram of pathways: (**a**) Venn diagram of pathways which are downregulated upon treatment and in the healthy control compared with inflamed control. (**b**) Venn diagram of pathways which are upregulated upon treatment and in the healthy control compared with inflamed control. Only genes with an adjusted *p*-value of *p* < 0.1 and FC > 1.5 are considered significant.

CM treatment resulted in the downregulation of 21 pathways (Figure 6a and Table 4). Of these, 8 pathways (38%) were unique to the CM treatment, while 12 (57%) were shared with the healthy control group and 1 (5%) with both the EV treatment and the healthy control group. Furthermore, 28 pathways were found to be upregulated only by CM treatment, with 17 (61%) of these pathways being unique to CM treatment and the remaining 11 (39%) being shared with the healthy control group (Figure 6b and Table 5).

In contrast, EV treatment upregulated no pathway and downregulated 3, of which 2 pathways (67%) were shared between EV treatment and the healthy control group and 1 (33%) with both the CM treatment and the healthy control group (Figure 6a and Table 4).

PF treatment did not significantly regulate any known pathway.

## 3. Discussion

Complete CM had the strongest treatment effect on inflamed tenocytes in our study. While all three treatments yielded significant differences compared with the untreated inflamed control, the EV fraction and the PF were not able to influence the gene expression level to the same extent as the CM fraction.

In line with our findings, the synergistic effects of the secretome’s EV and soluble protein components and the corresponding therapeutic superiority of complete CM compared with its subfractions, were shown in a variety of other cells and assays [53,54,59,60,61]. A comparison of the effects of CM and EV on adipose-derived MSCs (aMSCs) on muscle regeneration revealed that both CM and EV protect against cellular senescence but demonstrate higher proliferation and differentiation with CM, while only EVs reduced inflammation [53]. In an in vitro OA model comparing the therapeutic efficacy of human ASCs derived CM and EVs, both CM and EV reduced hypertrophic collagen 10, but only CM significantly decreased MMP activity and prostaglandin 2 (PGE2) expression [61]. Similarly, a study evaluating the effects of whole CM and its EV and PF fractions on inflamed nucleus pulposus and annulus fibrosus cells in vitro demonstrated a superior immunomodulatory and MMP inhibitory effect of whole CM compared with its subfractions [60]. A comparison of the effects of the peripheral blood mononuclear cell-derived secretome and its subfractions also found that the complete secretome induced better neo-angiogenesis than its apportioned constituents, namely the EV, lipid, and protein fractions individually [54].

In contrast, divergent findings were made by investigating the CM of human adipose-derived MSCs, where EVs alone, similar to the whole CM, were capable of promoting cell proliferation and migration in skeletal muscle cells. Furthermore, small extracellular EVs outperformed conditioned media of adipose tissue in migration and regeneration potential, although this study standardized EVs and CM on equivalent protein concentrations and did not consider unpacked proteins [61]. Another explanation for EVs outperforming CM on migration in this particular study is that the ability to recruit host cells for migration occurs more efficiently through EVs than through other paracrine factors that are present in the CM [62,63].

On the other hand, in a comparison of the therapeutic efficacy of amniotic MSCs derived whole CM, its PF, and EV fractions on immune cell proliferation and differentiation, whole CM and its PF fraction decreased peripheral blood mononuclear cell proliferation, reduced inflammatory polarization of T-cells, enhanced regulatory T-cell and M2 macrophage polarization, and reduced activation of B-cells, while EVs showed no immunomodulatory effect [59].

The divergent results of the various studies comparing of the therapeutic effects of complete CM and its EV fraction may be explained by the differences in the CM/EV donor cells, the media and substrates used for cell culture, cell confluence, preconditioning, the isolation and concentration procedures, the dosage and the treated cells [58,64,65,66,67,68].

Properties of CM/EV isolates are highly dependent on the donor cell type since different cell sources secrete various type of signals beneficial for alternating types of downstream applications [69,70]. In addition, isolation methods, especially for EVs, have an impact on the type and the properties of the enriched EVs [71,72]. Cell culture parameters such as seeding density or passaging frequency can further influence the regenerative potential [73,74].

In addition to the stem cell source, differences in secretome composition are significant when comparing fetal or adult donors [75,76]. Furthermore, pre-conditioning of the donor cells either with various compounds or by modification of the culture conditions leads to altered secretome profiles [77,78,79,80]. Another big obstacle when comparing CM and its subfraction is the standardization strategy for studies, the optimal dosage of the treatment, and the number of doses. CM is typically standardized based on the protein concentration, whereas the most common way to determine the therapeutic dose of an EV treatment is by quantifying the number of particles using NTA in an isolate. By using this method, the study focuses on all EVs, regardless of cargo. Future experiments are needed to assess the dose-dependent effects of all three fractions individually, with the aim of identifying the minimum effective dosage and confirming the superior therapeutic efficacy of complete CM compared with its fractions. An alternative approach is to focus on one type of cargo by quantifying a specific component such as nucleic acids, proteins, or lipids in the EVs or using Raman spectroscopy to ascertain the reproducibility of the CM/EV product composition for quality control [81].

Factors that are potentially beneficial for regeneration are distributed both within EVs and in the soluble fraction of the CM [82,83,84]. Furthermore, the presence of ribosomal proteins and the corresponding factors for translation in EVs would have a direct impact on the recipient cells by expressing proteins de novo [85]. Recently, proteomic analysis demonstrated diverging protein composition between the CM and the EVs, indicating that the vesicle-bound and soluble proteins differ, which confirms the necessity to analyze different parts of the secretome independently to get better insights into the course of events that take place upon administration of the CM and its subfractions before possible clinical use and also to have a clear understanding of protein expressions in healthy organisms and how they are altered by certain diseases with cell-free therapies in numerous clinical applications [43,44,45]. A better understanding of these processes would provide more information for potential tailored therapeutic options for the secretome.

This study, together with others, provides promising results in vitro. However, prior to translation into the clinic, several questions still have to be addressed, such as the route of administration of the agent, which is characterized by the absorption profile, the distribution throughout the body, the metabolism rate, and the elimination of the clearance [86]. In particular, clearance of EVs is a big problem since it has been shown that EVs, regardless of the route of administration, are quickly cleared from blood circulation and discharged from the body [87,88]. All these properties likely affect the therapeutic potential of the treatment. Upcoming studies are urgently needed that aim to resolve the issues mentioned above and pave the way for reproducible and optimized studies with cell-free treatment approaches.

This study is not without shortcomings. While we did standardize the EV or protein concentration within each treatment, it was not possible to standardize the EV or protein concentration between treatments as enriching one would affect the composition of the other and would ultimately cause loss of material. This issue is demonstrated by the fact that the protein concentration from the EV fraction and the PF combined does not equal the protein concentration of the CM (Figure 3a). In addition to that, the EV concentration in the EV fraction is lower than in the CM (Figure 3b). These observations can be explained by the filtering properties of the size exclusion chromatography (SEC) columns, which may lead to the loss of some EVs and proteins during the filtering process [89].

In conclusion, the differential gene expression data revealed that none of the three treatments was able to restore the healthy status of the patient cells following inflammation (Figure 3). However, treatment with the complete CM resulted in the highest number of differentially expressed genes relative to the inflamed control, suggesting a superior therapeutic effect compared with EV or PF treatment alone and a possible synergistic effect of the different secretome fractions.

## 4. Materials and Methods

### 4.1. Bone Marrow Collection and MSC Isolation

Bone marrow was harvested from three horses (donor 1: 11-year-old Tinker mare; donor 2: 6-year-old Pura Raza Espanola gelding; donor 3: 23-year-old Austrian Warmblood mare) euthanized for reasons unrelated to this study and without chronic diseases. Based on the “Good Scientific Practice, Ethics in Science and Research regulation” implemented at the University of Veterinary Medicine Vienna, the Institutional Ethics Committee of the University of Veterinary Medicine Vienna, and the Austrian Federal Ministry of Educations, Science, and Research, in vitro cell culture studies do not require approval if the cells were isolated from tissue that was obtained either solely for diagnostic or therapeutic purposes or in the course of other institutionally and nationally approved experiments.

Immediately post-mortem, bone marrow was harvested from the sternum under sterile conditions, as we previously described [58,90].

The aspirated bone marrow was mixed with 1× PBS with Mg/Ca (Life Technologies, UK, shipped from NL, PBS+/+, Gibco, 1404009) (1:1) and filtered through a 100 µM cell strainer (Greiner Bio-One GmbH, Germany, shipped from Germany, 542000). The mononuclear cell fraction was isolated by density gradient centrifugation using Ficoll Paque Premium (Cytiva, Sweden, shipped from France, 11743219) as previously published [58,90,91,92]. In brief, the collected bone marrow—PBS +/+ mix was layered onto the Ficoll and centrifuged at room temperature for 30 min at 300× *g* (Hettich, Germany, shipped from Germany, Rotanta 460R) without brake. The buffy coat was collected and washed once with PBS +/+ by centrifuging with 300× *g* for 5 min. The obtained mononuclear cells were seeded in DMEM (Gibco, 31885023) supplemented with 10% FCS (Sigma Aldrich US, shipped from Germany, F7524), 1% Pen/Strep (Sigma, P433-100 mL), and 1% Amphotericin B (Biochrom Germany, shipped from Germany, A 2612-50 mL) and cultured in an incubator with 20% O_2_ and 5% CO_2_. The medium was changed every 2-3 days. Isolated MSCs were characterized according to the criteria defined by the International Society for Cellular Therapies: plastic adherence, expression of surface markers CD90, CD44, and CD29, lack of expression of CD31, Pan B and IgG, and adipogenic, chondrogenic, and osteogenic trilineage differentiation potential. After reaching 80–90% confluency, MSCs were harvested and frozen until further use.

### 4.2. Isolation of the Conditioned Medium (Fractions), Characterization, and Quantification

MSCs were thawed and expanded in chemically defined complete medium (DMEM) supplemented with 10% FCS (Capricon Scientific, Germany, shipped from Germany, FBS-12A), 1% Pen/Strep, and 1% amphotericin B until 80–90% confluency, passaged with trypsin (Gibco, 25300096), and seeded at a defined density of 4 × 10^6^ cells per T175 flask (Sarstedt, Nümbrecht, Germany, 833912002). All cells used were at passage 2, cell morphology was assessed daily, and viability was measured using Trypan blue dye exclusion in conjunction with an automated cell counter (ThermoFisher Countess 3). To ensure that regenerative effects originate exclusively from the MSCs secretome and not from culture supplements and to avoid the confounding factor of the inherent batch-to-batch variability of serum [93,94,95]. MSCs were washed twice with 10 mL filtered (0.22 µm filter (Sarstedt, Nümbrecht, Germany, shipped from Germany, 831822)) PBS +/+ after 24 h and then cultured under serum-free conditions without antibiotics (DMEM with 1 g/L Glucose, L-Glutamine, 110 mg/L Sodium Pyruvate w/o Pen/Strep, FCS, and Amphotericin B)). After 48 h, a total volume of 60 mL of the conditioned medium (CM) was collected, transferred into 50 mL Falcon tubes (Sarstedt, 64547254), and pre-centrifuged at 3000× *g* for 20 min at 4 °C to remove undesired cell debris. After the centrifugation, the CM was used without further concentration steps at its original concentration. The CM was divided into thirds; one third was used as full CM, and the other two-thirds were immediately loaded onto qEV10/35 nm (IZON, qEV10 35 nm) columns, which have an optimal recovery range of 35 nm to 350 nm according to the manufacturer’s protocol. The eluted EVs from fractions 2 and 3 were pooled and served as the extracellular vesicle (EV) fraction. Fractions number 10 and 11 contain a high rate of proteins and were used as the protein fraction (PF). Fractions 1, 4–9, 12, and higher were discarded. CM, EVs, and PF were stored at 4 °C and used within 3 h.

Nanotracking Analysis (NTA) was performed to measure the size distribution and quantity of the isolated particles in scatter mode with a 488 nm laser. The minimum brightness was set to 30, the minimum area to 10, the maximum area to 1000, the maximum brightness to 255, the shutter to 400 and the temperature to 25 °C. Isolates were measured in technical triplicates. Fluorescence-triggered flow cytometry (FT-FC)) was used to characterize the EVs and evaluate their size distribution as described previously [58,96]. Western blot against CD9 and CD63 and transelectron microscopy were carried out as we previously described [58].

Protein concentration was measured with the Qubit Protein Assay Kit (ThermoFisher, Q33211) using the Qubit 4 Fluorometer (Thermo Fisher Scientific, Singapore, shipped from Germany) according to the manufacturer’s protocol. In brief, a working solution was prepared by diluting the Qbit reagent 1:200 in Qubit buffer (190 µL) for each sample (10 µL). The samples and the protein standards were mixed with the working solution 1:20 and incubated for 15 min light protected at room temperature. Finally, the samples were read in the device.

For all three treatments, the concentration of protein and EVs was standardized to the lowest measurements for each fraction. The EV fractions were standardized to contain 1 × 10^9^ EVs/mL and an optimal size range between 35 nm and 350 nm based on the used isolation columns. The PF was standardized to contain 72 µg/mL protein without any detectable EVs. We chose to normalize the CM fraction based on the lowest measured protein concentration (118 µg/mL containing 1.365 × 10^11^ particles/mL ± 1.43 × 10^10^) rather than on the EV concentration because the difference between the protein content of the CM and the PF was larger than the differences in the EV concentration between the CM and the EV fraction (Figure 2a).

### 4.3. Tenocyte Isolation

Tendon samples were obtained from the mid-metacarpal region of the superficial digital flexor tendon of the same three horses as already described by our group [58,97]. In brief, the paratenon was removed under sterile conditions, and the tendons were sectioned into small pieces (<0.5 × 0.5 × 0.5 cm). Isolation of cells was performed by migration from explants in DMEM supplemented with 10% FCS, 1% Pen/Strep, and 1% Amphotericin B in an incubator with 20% O_2_ and 5% CO_2_ (explants were removed after 7–10 days). Cells were expanded until 80–90% confluency before passage.

### 4.4. Inflammation and Treatment Strategy

All in vitro experiments were performed with three biological replicates (three donors) and three technical replicates 400,000 tenocytes were seeded per well on 12-well plates and cultured in DMEM supplemented with 10% FCS, 1% Pen/Strep, and 1% Amphotericin B in an incubator with 20% O_2_ and 5% CO_2_ for 48 h. After 48 h, tenocytes were rigorously washed twice with filtered PBS +/+ and divided into 5 groups: a healthy control group, an inflamed untreated control group, and three inflamed treated groups with CM, EV, or PF treatment (time 0). As serum starvation induces an inflammatory response, the healthy control group was cultured in DMEM with 1 g/L Glucose, L-Glutamine, 110 mg/L Sodium Pyruvate w/o Pen/Strep, FCS and Amphotericin B with 20% EV-depleted FCS (FCS was depleted using Amicon Ultra 15, (Merck Millipore Ltd, Irland, shipped from Germany, UFC910024) 3000 g for 55 min at 4 °C) to provide the minimum amount of nutrients while simultaneously reducing the compromising effect of too high serum levels on the phenotypic drift in tenocytes, which is hallmarked by the reduction of tendon marker genes such as Scx, Mkx, and collagen subtypes, and thus ensuring the health of the control group [98,99,100,101,102]. Tenocytes of the inflamed groups were cultured in serum-and antibiotic-free medium (DMEM with 1 g/L Glucose, L-Glutamine, 110 mg/L Sodium Pyruvate), and inflammation was induced by serum starvation plus chemical stimulation with 10 ng/mL TNFα (ImmunoTools, Germany, shipped from Germany, 11343013) and 10 ng/mL ILb1 (ImmunoTools, Germany, shipped from Germany, 103010501) for 24 h. After 24 h (time 24 h), half (500 µL) of the inflamed serum– and antibiotic-free medium (1 mL) was removed, and 1 mL of treatment (autologous CM, EVs, or PF) was added. The inflamed controls received 1 mL of fresh inflamed serum and antibiotic-free media, and the healthy controls received 1 mL of fresh medium with 20% EV-depleted FCS. After 24 h (time 48 h), the tenocytes of all treatment and control groups were harvested for mRNA sequencing.

### 4.5. Flow Cytometry

Cells were trypsinized at passage 2. Subsequently, 1 × 10^5^ cells per sample were washed with PBS supplemented with 2% FCS. The flow cytometry analysis utilized the following monoclonal antibodies and their respective isotype controls: PE-CD29 (Clone TS2/16, Mouse IgG, 1:50, Biolegend, San Diego, CA, USA), FITC-CD31 (Clone CO.3E1D4, IgG2a, 1:50, Biorad, Hercules, CA, USA), FITC-CD44 (Clone 25.32, IgG, 1:50, Biorad), Purified CD90 (Clone DH24A, IgM, 1:50, Monoclonal Antibody Center), FITC-PanB cells (Clone CVS36, IgG1, 1:50, Biorad). A total of 1 × 10^4^ events were measured per sample.

### 4.6. Trilineage Differentiation

Experimental differentiation procedures were conducted in triplicates (passage 2), and the cultures were maintained for a duration of three weeks. The cultural media were refreshed every three days. The control samples were cultured in DMEM supplemented with 10% FCS, 1% Pen/Strep, and 1% amphotericin B. Adipogenic and osteogenic differentiations were performed by seeding 4000 MSCs per well of a 12-well plate in DMEM supplemented with 10% FCS, 1% Pen/Strep, and 1% amphotericin B for 48 h. Subsequently, the cells were washed with PBS and treated with either adipogenesis ((ThermoFisher, Waltham, MA, USA, A1007001)) or osteogenesis (ThermoFisher, A1007201) differentiation media. For chondrogenic differentiation, 350,000 MSCs were collected in 15 mL Falcon tubes and pelleted, followed by resuspension in chondrogenesis (ThermoFisher, A1007101) differentiation media.

#### 4.6.1. Oil Red Staining

A solution was prepared by diluting six parts of Oil red O (Sigma, O0625-25G) in four parts of distilled water. The mixture was allowed to mix overnight at room temperature and subsequently filtered. The cells were fixed using 60% isopropanol (Riedel de Haen, Seelze, Germany, 24137) for 5 min, followed by incubation with the prepared Oil red O solution for 10 min. Finally, the differentiated cells were washed with 60% isopropanol and distilled water, counterstained with haematoxylin (Merck, Kenilworth, NJ, USA, 108562), and washed again with distilled water.

#### 4.6.2. Alcian Blue Staining

Paraffin-embedded samples of chondrocyte pellets were sectioned at a thickness of 3 μm using a microtome (CUT2511A, MicroTec, Brixen, Italy). The paraffin blocks were precooled at −15 °C and cut into sections, which were then transferred to cold water using wet brushes and subsequently transferred to warm water at 40 °C with nonadhesive standard microscope slides. The sections were flattened out and collected on super frost adhesive microscope glass slides. The slides were labeled and allowed to dry overnight at room temperature. Following this, the slides were heated at 60 °C for 20 min in an incubator, submerged in xylene twice for 10 min at room temperature, and incubated in 100% isopropanol and decreasing concentrations of ethanol (96%, 70%, and 50%) for 5–10 min each. Subsequently, the slides were washed with distilled water and stained with haematoxylin for 3 min. Finally, the slides were washed with distilled water, stained with eosin for 30 s, dehydrated in ethanol, and mounted using Aquatex mounting medium (Merck Millipore, Burlington, MA, USA). The slides were then dried overnight and analyzed using the FL Auto Imaging System (Invitrogen (Waltham, MA, USA), EVOS (Life Technologies, Bothell, WA, USA, shipped from Germany)).

#### 4.6.3. Von Kossa Stain

The cells were fixed with 5% formaldehyde (Sigma), followed by incubation with 5% silver nitrate (Carl Roth, Austria, shipped from Austria, 9370.9) under UV light for 20 min. Afterward, the cells were washed with distilled water, fixed with 5% sodium thiosulfate, and washed again with distilled water. Subsequently, the cells were stained with nuclear fast red (Waldeck GmbH, Germany, shipped from Germany, 221833) for 10 min and washed once more.

### 4.7. mRNA-Sequencing of the “Patient Cells” (Tenocytes)

Due to cost considerations, we combined the technical replicates for RNASeq and ran one sample for each biological replicate (donor) per treatment/control group. The overall quality of the next-generation sequencing data was evaluated automatically and manually with fastQC v0.11.8 and multiQC v1.7 [103,104]. Reads from all passing samples were adapter-trimmed and quality-filtered using bbduk from the bbmap package v38.69 and filtered for a minimum length of 17 nt and phred quality of 30. Alignment steps were performed with STAR v2.7 using samtools v1.9 for indexing, whereas reads were mapped against the genomic reference GRCm38.p6 provided by Ensembl (Cambridge, UK) [105,106,107]. Assignment of features to the mapped reads was completed with htseq-count v0.13 [108]. Differential expression analysis with edgeR v3.30 used the quasi-likelihood negative binomial generalized log-linear model functions provided by the package [109]. The independent filtering method of DESeq2 was adapted for use with edgeR to remove low-abundant genes and thus optimize the false discovery rate (FDR) correction [110]. RT-qPCR validation was not used in this study due to the well-established robust nature of RNAseq methods [111,112].

### 4.8. Data Analysis

We selected a less stringent *p*-value cutoff of 0.1 in our analysis to avoid missing potentially relevant genes with subtle but biologically significant expression changes. By employing a more inclusive approach, we enhanced the sensitivity of our analysis, explored novel insights, accounted for biological complexity, addressed sample size limitations, and facilitated integration with other datasets. We ensured statistical rigor while capturing valuable findings beyond a stricter cutoff. RNASeq data were read into the R statistical environment and processed using the DESeq2 package [110]. Statistical analysis of preprocessed NGS data was completed with R v3.6 and the packages pheatmap v1.0.12, pcaMethods v1.78, and genefilter v1.68. Differential expression analysis with edgeR v3.28 used the quasi-likelihood negative binomial generalized log-linear model functions provided by the package [109]. The independent filtering method of DESeq2 was adapted for use with edgeR to remove low-abundance mRNAs and thus optimize the false discovery rate (FDR) correction [110]. To determine differentially expressed genes, a linear mixed model with subject ID as a random effect was chosen. Significant differential expression (DE) was assumed for adjusted *p*-values < 0.1 and a fold change (FC) > 1.5. Results were put into a biological context using gene set variation analysis with the molecular signature database, Wiki and KEGG pathways as input [113,114,115,116]. Significant differences between GSVA scores were determined using LIMMA and the linear mixed model as described above [117].

Principle component analysis (PCA) was used to assess the clustering of samples based on treatment groups. The statistical analysis, a repeated measures ANOVA, was performed using Graph Pad Prism v.6.01 (GraphPad Software, San Diego, CA, USA). The number of used donors (*n*), the *p*-values and the respective statistical significance are indicated in each figure. The data are plotted as mean with ± standard error of mean in scatter plots and ±standard deviation in bar graphs.

Venn diagrams and volcano plots were generated using online tools (InteractiveVenn and VolcaNoseR) [118,119].

## Figures and Tables

**Table 1:** Measured protein- and particle-concentration per fraction: The CM fraction contained the highest protein content (µg/mL) followed by the PF. The lowest protein concentration was found in the EV fraction. The highest overall particle concentration (particles/mL) was found in the CM which is also true for the particle numbers >195 nm. However, more particles were enriched in the EV fraction ≤195 nm. No measurable particles were present in the PF.

	CM	EV	PF
Protein (µg/mL)	130 ± 6.18	30.4 ± 0.5	73.2 ± 0.9
Particle number (total NTA) (particles/mL)	1.53 × 10^11^ ± 2.01 × 10^10^	1.03 × 10^11^ ± 2.04 × 10^10^	Not detected
Particle number ≤ 195 nm (NTA) (particles/mL)	5.36 × 10^10^ ± 7.67 × 10^9^	5.61 × 10^10^ ± 1.06 × 10^10^	Not detected
Particle number > 195 nm (NTA) (particles/mL)	1.09 × 10^11^ ± 2.51 × 10^10^	4.67 × 10^10^ ± 9.81 × 10^9^	Not detected

**Table 2:** Top 10 down-regulated genes of the three treatment groups (conditioned medium (CM), extracellular vesivles (EV) and soluble protein fraction (PF)) and the healthy control (CRTL) compared with the inflamed CTRL: Only genes with an adjusted *p*-value of *p* < 0.1 and FC > 1.5 are considered significant.

Top 10 Downregulated Genes in the Treatment/Healthy CTRL vs. Inflamed CTRL (Sorted by Ascending Adjusted *p*-Value)											
CM			EV			PF			Healthy CTRL		
Gene	FC	Adj. *p*-Value	Gene	FC	Adj. *p*-Value	Gene	FC	Adj. *p*-Value	Gene	FC	Adj. *p*-Value
ADGRD1	−23.750	9.5536 × 10^−7^	FUZ	−36.203	3.0566 × 10^−16^	NOXA1	−40.887	6.1398 × 10^−20^	PSME2	2.11877	2.5995 × 10^−40^
SAA	−31.261	5.7985 × 10^−6^	NTN1	−30.152	0.00027148	TRIM6	−34.742	3.7594 × 10^−17^	KYAT3	1.86235	2.2722 × 10^−35^
HIVEP3	−15.446	1.0763 × 10^−5^	GPM6B	−29.649	0.00027148	H1-9	−30.859	4.3641 × 10^−8^	RAB20	3.90099	2.2722 × 10^−35^
COL14A1	−28.138	9.163 × 10^−5^	podocalyxin like	−28.229	0.01057581	TCP11	−33.531	1.2429 × 10^−5^	DBNL	2.24989	9.5604 × 10^−34^
ZIC2	−12.980	0.00011	LRRC23	−27.298	0.01865918	MRPL54	−31.808	2.4525 × 10^−5^	SNAP29	1.72103	5.1924 × 10^−32^
VSIR	−17.357	0.00038	C6orf47	−21.264	0.03612666	ZNF382	−17.616	5.3998 × 10^−5^	CCL2	4.07345	3.5555 × 10^−30^
FBXO32	−16.372	0.00056	CMYA5	−18.940	1.5926 × 10^−7^	FAM216B	−16.926	0.00018	CCL11	3.49896	6.2286 × 10^−30^
PLA2G2A	−26.155	0.00062	ZKSCAN8	−17.918	0.00027148	NTN1	−29.748	0.00031	PI3	3.77388	2.2449 × 10^−29^
SCARA5	−43.596	0.00067	CSPG4	−17.519	1.3391 × 10^−5^	CCDC40	−26.832	0.00257	CCL5	5.21572	4.8974 × 10^−29^
SPNS3	−31.756	0.00205	GSK-3	−17.2511	0.00031	IL17RA	−17.499	0.00443	SIDT2	3.70467	3.1246 × 10^−27^

**Table 3:** Top 10 up-regulated genes of the three treatment groups (conditioned medium (CM), extracellular vesivles (EV) and soluble protein fraction (PF)) and the healthy control (CRTL) compared with the Inflamed CTRL: Only genes with an adjusted *p*-value of *p* < 0.1 and FC > 1.5 are considered significant.

Top 10 Upregulated Genes in the Treatment/Healthy CTRL vs. Inflamed CTRL (Sorted by Ascending Adjusted *p*-Value)											
CM			EV			PF			Healthy CTRL		
Gene	FC	Adj. *p*-Value	Gene	FC	Adj. *p*-Value	Gene	FC	Adj. *p*-Value	Gene	FC	Adj. *p*-Value
PIRT	186.866	9.78 × 10^−10^	ZNF582	443.953	7.7528 × 10^−46^	ZNF582	432.963	9.88 × 10^−43^	ZNF582	−45.537	8.3178 × 10^−50^
ELMO1	181.622	3.61 × 10^−10^	PIRT	197.946	6.2092 × 10^−12^	PIRT	186.640	3.19 × 10^−10^	ENSECAG00000016730	−28.492	5.4407 × 10^−42^
ZNF501	239.838	3.61 × 10^−7^	ELMO1	173.926	1.6926 × 10^−6^	CCND1	198.841	3.53 × 10^−7^	CCDC88A	−27.159	1.195 × 10^−32^
ENSECAG00000023978	184.840	1.75 × 10^−6^	ZNF501	226.906	1.9717 × 10^−6^	metallothionein-1A	451.660	3.53 × 10^−7^	CELF2	−28.702	6.2556 × 10^−29^
TPGS2	173.051	4.13 × 10^−6^	ENSECAG00000023978	174.590	1.4739 × 10^−5^	TPGS2	178.046	8.18 × 10^−7^	FSTL1	−32.601	1.8517 × 10^−25^
ENSECAG00000013505	182.588	7.55 × 10^−6^	ZNF182	286.245	1.6141 × 10^−5^	ELMO1	167.381	4.86 × 10^−6^	PTMA	−26.727	1.2088 × 10^−21^
STAC	215.231	3.03 × 10^−5^	ENSECAG00000013505	160.195	0.00035	ZNF182	292.836	7.23 × 10^−6^	CALCA	−47.284	2.7129 × 10^−21^
CCND1	163.745	0.00012	DCX	205.337	0.00060	ZNF501	214.091	1.24 × 10^−5^	C7H11orf58	−29.221	4.2637 × 10^−20^
RAD51D	210.350	0.00038	ASB5	161.541	0.00447	MT1B	163.702	0.00018	ANXA5	−41.201	2.5081 × 10^−19^
ASB5	176.871	0.00038	GRID1	273.429	0.01865	ACBD7	319.023	0.00020	APC	−17.720	1.6149 × 10^−17^

**Table 4:** Down-regulated KEGG pathways in the treatment groups conditioned medium (CM) and extracellular vesicles (EV) compared with the inflamed CTRL. Protein fraction (PF) treatment did not significantly regulate any known pathway. Only pathways with adjusted *p*-value < 0.1 are considered significant.

Downregulated Pathways					
CM			EV		
Pathway	FC	Adj. *p*-Value	Pathway	FC	Adj. *p*-Value
Secretion of hydrochloric acid in parietal cells	−1.1589254	0.09355804	Glycerolipids_and_glycerophospholipids	−0.6118861	0.06337278
Nephrogenesis	−1.0681281	0.01124854	Myd88 distinct input/output pathway	−0.5034747	0.06337278
Fatty acid omegaoxidation	−1.0049143	0.07476631	Autosomal recessive osteopetrosis pathways	−0.5445	0.06826365
Complement activation	−0.7625858	0.07859295			
Interleukin1 induced activation of nfkb	−0.674886	0.03094377			
Tryptophan metabolism	−0.6162714	0.04539786			
Mammalian disorder of sexual development	−0.6081617	0.03202702			
Biomarkers for urea cycle disorders	−0.5883797	0.05081052			
Cysteine and methionine catabolism	−0.5820551	0.04634927			
Oxidative stress response	−0.5797825	0.09005984			
Eicosanoid synthesis	−0.5773651	0.03997058			
Overview of nanoparticle effects	−0.5647847	0.03762172			
Beta alanine metabolism	−0.5222863	0.09005984			
Matrix metalloproteinases	−0.4351830	0.07476631			
S1P receptor signal transduction	−0.4120393	0.05402778			
Autosomal recessive osteopetrosis pathways	−0.4056264	0.07915106			
Valine leucine and isoleucine degradation	−0.3839604	0.08790938			
Sarscov2 innate immunity evasion and cellspecific immune response	−0.3664584	0.04539786			
Selenium micronutrient network	−0.3640307	0.05698454			
Onecarbon metabolism and related pathways	0.31911033	0.08974443			
Leukocyte transendothelial migration	−0.2761994	0.07915106			

**Table 5:** Up-regulated KEGG pathways in the treatment group conditioned medium (CM) compared with the inflamed CTRL. Treatment with extracellular vesicles (EV) and protein fraction (PF) did not significantly regulate any known pathway. Only pathways with adjusted *p*-value < 0.1 are considered significant.

Upregulated Pathways		
CM		
Pathway	FC	Adj. *p*-Value
Nucleotide excision repair in xeroderma pigmentosum	0.3494815	0.07130188
Pyrimidine metabolism	0.37184491	0.08655644
Dna repair pathways full network	0.40690974	0.04539786
Oocyte meiosis	0.40797081	0.03997058
Nucleotide excision repair (WP)	0.42922411	0.03776133
Nucleotide excision repair (KEGG)	0.42922411	0.03776133
Progeriaassociated lipodystrophy	0.4650666	0.04539786
Pyrimidine metabolism	0.47735464	0.03202702
Cell cycle (WP)	0.49283852	0.05915343
Cell cycle (KEGG)	0.4933381	0.05577275
Gastric cancer network 1	0.49568256	0.09520106
DNA irdamage and cellular response via ATR	0.50092208	0.04101562
Base excision repair	0.55896011	0.09005984
Cohesin complex cornelia de lange syndrome	0.58300193	0.01124854
Mammary gland development pathway puberty stage 2 of 4	0.58799809	0.04539786
Base excision repair	0.59293411	0.09955858
Gastric cancer network 2	0.67347864	0.04101562
ATR signaling	0.68398751	0.07272085
Homologous recombination	0.70623154	0.04539786
Retinoblastoma gene in cancer	0.71687919	0.03776133
Nucleotide metabolism	0.76157908	0.04101562
Serine metabolism	0.76326554	0.01124854
Mismatch repair	0.81963998	0.03202702
Regulation of sister chromatid separation at the metaphaseanaphase transition	0.86498193	0.03856315
DNA replication	0.904723	0.03094377
DNA mismatch repair	0.92494463	0.03202702
Acquired partial lipodystrophy barraquersimons syndrome	0.93639734	0.03202702
DNA replication	0.96512563	0.03202702

## Data Availability

The data presented in this study are available on request from the corresponding author.
